# Awareness and uptake of genetic testing among individuals at-risk for hereditary colon cancer

**DOI:** 10.1186/1897-4287-9-S1-P22

**Published:** 2011-03-10

**Authors:** Jan T Lowery, Al Marcus, Nora Horick, Dianne Finkelstein, Dennis J Ahnen

**Affiliations:** 1 University of Colorado, Colorado School of Public Health, Aurora, CO, USA; 2 Massachusetts General Hospital, Boston, MA, USA; 3 Veterans Administration Medical Center, Denver, CO, USA; 4 University of Colorado School of Medicine, Aurora CO, USA

## Background

Referral for genetic counseling and testing is appropriate for individuals whose families meet clinical criteria for Hereditary Non-Polyposis Colorectal Cancer (HNPCC) and for some families with members that meet the Bethesda criteria. The FHPP is a prospective randomized trial to evaluate an intervention to promote colonoscopy in members of high risk CRC families. FHPP participants were surveyed to assess their knowledge about and utilization of genetic testing for HNPCC.

## Methods

Unaffected family members of CRC cases were recruited into FHPP from the Colon Family Registry and the Cancer Genetics Network. Surveyed participants were classified either as HNPCC if their families met Amsterdam II criteria (n=91) or high-risk (HR) if their families did not meet Amsterdam but met the Bethesda criteria of one first degree relative with CRC under age 50 (n=161).

## Results

Sixty-percent of participants were female, 30% were <50 yrs and 94% had a regular doctor. Nearly 90% had discussed their family history of CRC with their doctor. Fifty-five percent of participants had heard of genetic testing; 25% from their doctor or other provider, 21% from family, 32% from media, and 19% from participation in FHPP. HNPCC participants were more likely than HR to have heard about genetic testing (70% vs. 46%, p<0.001) and to have discussed testing with their doctors (34% vs. 16%, p=0.03). Thirty percent of participants had been advised to consider genetic testing (33% of HNPCC, 15% of HR but only 20% of the HNPCC and 4% of the HR participants had had genetic testing. The most common reasons cited for not having testing were: not advised (30%), too busy (32%) and cost (7%); 31% were still considering testing. Among participants that were tested or knew that a family member had been tested (n=50), 80% said they had discussed results with other family members. However, 25% of participants did not know whether their family members had had testing and 38% did not know the results of their family member’s gene test.

## Conclusions

The low level uptake of genetic testing in the FHPP high risk populations appears to be due to a lack of awareness of the importance of genetic testing by both the participant and their provider and a reticence on the part of the participant to proceed with genetic testing if it is advised. Improving patient-provider education and family communication about genetic testing may help to increase utilization of appropriate genetic services.

